# Intelligence

**Published:** 1821-09-01

**Authors:** 


### INTELLIGENCE.

The Proprietor of the American Medical Recorder, Mr. Webster, of Philadelphia is republishing, in the United States, the following British works, viz.—

1. Mr. Cooper's Surgical Dictionary (3d edition.)
2. Dr. Johnson on Derangements of the Liver, *(from the 3d edition.)*
3. Dr. Parry's Elements of Pathology.
4. Messrs. Cooper and Travers' Surgical Essays.
5. Dr Parry (Sen.) on the Pulse.
6. Mr. Lawrence on Hernia.
7. Dr. Johnson's Influence of Tropical Climates on European Constitutions (*3d edition.)*

---

We understand that a Course of Lectures on Medical Jurisprudence will be given at Glasgow, by Dr. Kennedy, during the ensuing winter.

---

A Young Gentleman, 24 years of Age, educated in Scotland, and possessing a Surgical Diploma from Glasgow, is desirous of a Situation as an Assistant to a General Practitioner.

☞ *The Editor can take upon himself to recommend the Gentleman in question, as a very desirable acquisition, where steadiness, good moral character, and respectable professional acquirements, are looked for. A line, (post paid) to Dr. Johnson, will be attended to.*

---

Dr. Allan Maclean, of Caiu's College, Cambridge, son of Sir L. Maclean, M. D. of Sudbury, was elected Physician to the Essex and Colchester Hospital, on the 31st May, in the room of Dr. Beatty, resigned.

---

☞ *The Lists of new Subscribers from Sudbury, Wincanton, &c. were too late for insertion in this number. The names will appear in our next.*

